# Functional genomics of the horn fly, *Haematobia irritans *(Linnaeus, 1758)

**DOI:** 10.1186/1471-2164-12-105

**Published:** 2011-02-10

**Authors:** Lorena Torres, Consuelo Almazán, Nieves Ayllón, Ruth C Galindo, Rodrigo Rosario-Cruz, Héctor Quiroz-Romero, José de la Fuente

**Affiliations:** 1 Facultad de Medicina Veterinaria y Zootecnia, Universidad Autónoma de Tamaulipas, Km. 5 carretera Victoria-Mante, CP 87000 Ciudad Victoria, Tamaulipas, Mexico; 2 Instituto de Investigación en Recursos Cinegéticos IREC (CSIC-UCLM-JCCM), Ronda de Toledo s/n, 13005 Ciudad Real, Spain; 3 Centro Nacional de Investigación Disciplinaria en Parasitología Veterinaria, Carretera Federal Cuernavaca-Cuautla 8534, Col Progreso, Jiutepec, Morelos. CP 62550, Mexico; 4 Departamento de Parasitología, Facultad de Medicina Veterinaria y Zootecnia, Universidad Nacional Autónoma de México, Ciudad Universitaria, Coyoacán, D.F. CP 04510, Mexico; 5 Department of Veterinary Pathobiology, Center for Veterinary Health Sciences, Oklahoma State University, Stillwater, OK 74078, USA

## Abstract

**Background:**

The horn fly, *Haematobia irritans *(Linnaeus, 1758) (Diptera: Muscidae) is one of the most important ectoparasites of pastured cattle. Horn flies infestations reduce cattle weight gain and milk production. Additionally, horn flies are mechanical vectors of different pathogens that cause disease in cattle. The aim of this study was to conduct a functional genomics study in female horn flies using Expressed Sequence Tags (EST) analysis and RNA interference (RNAi).

**Results:**

A cDNA library was made from whole abdominal tissues collected from partially fed adult female horn flies. High quality horn fly ESTs (2,160) were sequenced and assembled into 992 unigenes (178 contigs and 814 singlets) representing molecular functions such as serine proteases, cell metabolism, mitochondrial function, transcription and translation, transport, chromatin structure, vitellogenesis, cytoskeleton, DNA replication, cell response to stress and infection, cell proliferation and cell-cell interactions, intracellular trafficking and secretion, and development. Functional analyses were conducted using RNAi for the first time in horn flies. Gene knockdown by RNAi resulted in higher horn fly mortality (protease inhibitor functional group), reduced oviposition (vitellogenin, ferritin and vATPase groups) or both (immune response and 5'-NUC groups) when compared to controls. Silencing of ubiquitination ESTs did not affect horn fly mortality and ovisposition while gene knockdown in the ferritin and vATPse functional groups reduced mortality when compared to controls.

**Conclusions:**

These results advanced the molecular characterization of this important ectoparasite and suggested candidate protective antigens for the development of vaccines for the control of horn fly infestations.

## Background

The horn fly, *Haematobia irritans *(Linnaeus, 1758) (Diptera: Muscidae) is one of the most important ectoparasites of pastured cattle [1]. This fly was originally introduced from Europe and currently represents a tremendous health problem for cattle in the Americas from Southern Canada to Argentina [2]. Although horn flies parasitize mainly cattle, occasionally they feed on horses, sheep and dogs [3].

The developmental cycle of *H. irritans *is very short, taking from 10 to 14 days to complete. Larvae and pupae develop on dung and once the flies emerge from pupae, immediately start and remain feeding on cattle during their whole life. Flies leave the host only to move to others or to lay eggs on fresh manure [1]. Both males and females feed 24 to 38 times per day ingesting an average of 14.3 mg blood per fly [4].

Horn flies infestations interfere with animal feeding, thus producing significant reductions in weight gain and milk production [5,6]. The economic impact of *H. irritans *on livestock in the United States was estimated in approximately US$1 billion annually [7,8]. In dairy cattle, infestations higher than 200 flies per animal produce a loss of 520 ml milk and 28 kg weight daily [6]. In beef cattle, *H. irritans *infestations can cause a reduction of 8.1 kg weight daily [5]. Moreover, the skin lesions caused by the intermittent feeding of horn flies produce significant hide damages, affecting considerably the leather industry [9]. Additionally, horn flies are mechanical vectors of different pathogens that cause disease in cattle [10-14].

The control of horn flies has been primarily based on the use of chemical insecticides [15,16]. This control strategy has been partially successful but has resulted in the selection of flies resistant to most commercially available insecticides [15-17]. In addition to resistance, chemical insecticides affect other living organisms, contribute to environmental pollution and contaminate cattle products for human consumption.

Recently, research has been conducted to develop new horn fly control strategies that are cost-effective and environmentally friendly. The efficacy of the entomopathogenic fungi, *Metarhizium anisopalinae*, against horn fly larvae was very high in vitro [18]. However, field application of entomopathogenic fungi for biological control of horn flies is difficult. The use of female-specific conditional lethality systems has been also considered but not yet developed [19].

The immunological control of ectoparasite infestations was demonstrated through cattle vaccination against tick infestations [20,21]. The effect of anti-tick vaccines on the reduction of cattle tick infestations and the transmission of some tick-borne pathogens [21-23] and preliminary results obtained in insect vector species [24-32] have provided evidence that protective antigens may be used for development of vaccines with the dual target control of both arthropod infestations and reduction of vector capacity to transmit pathogens that impact human and animal health. Recently, Cupp et al. [33] demonstrated that horn flies fed on cattle immunized with the anti-clotting factor thrombostasin, took smaller blood meals and the egg development was delayed. Although other molecules have been proposed as vaccine candidates against horn flies [16,34,35], further research is needed to identify new vaccine candidates for effective control of horn fly infestations.

Recently, RNA interference (RNAi) was proposed as a method to identify candidate tick protective antigens [36] and was used for the screening of tick genes with potential applications in vaccine development [37-39].

The aim of this study was to conduct a functional genomics study in female horn flies using Expressed Sequence Tags (EST) analysis and RNAi. The results of this study will advance the molecular characterization of this important ectoparasite and suggested candidate protective antigens for the development of vaccines for the control of horn fly infestations.

## Results

### Assembly and annotation of female horn fly Expressed Sequence Tags (ESTs)

A cDNA library was made from whole abdominal tissues collected from partially fed adult female horn flies. From 2,462 sequenced ESTs, 302 and 2,160 were low and high quality ESTs, respectively (Table 1). Empty or vector ESTs were not obtained.

**Table 1:** Statistics of horn fly EST assembly.

Number of sequences	2,462
Mean length ± S.D. before vector stripping	877 ± 44 bp

Mean length ± S.D. after vector stripping	653 ± 32 bp

High quality EST reads	2,160

Assembled ESTs	2,160

No. unigenes	992

Mean length ± S.D.	758 ± 46 bp

No. unigenes with more than 20 ESTs	13

No. unigenes with 5-20 ESTs	44

No. unigenes with less than 5 ESTs	935

No. contigs	178

No. singlets	814

Novelty (unigenes/assembled ESTs)	46%

Redundancy (1-Novelty)	54%

Since the female horn fly cDNA library was not normalized, the EST distribution per contig was quantified to determine the redundancy level of our EST dataset. High quality ESTs were assembled into 992 unigenes (178 contigs and 814 singlets) (Table 1; Additional file 1: Table S1), representing 46% novelty (unigenes/assembled ESTs) in our dataset. ESTs (814) present as singleton sequences represented 82% of all unigenes, while 72 unigenes (7%) contained only two ESTs. On average, the number of ESTs per unigene was 2.2, which suggested a low diversity in our dataset.

**Figure 1:**
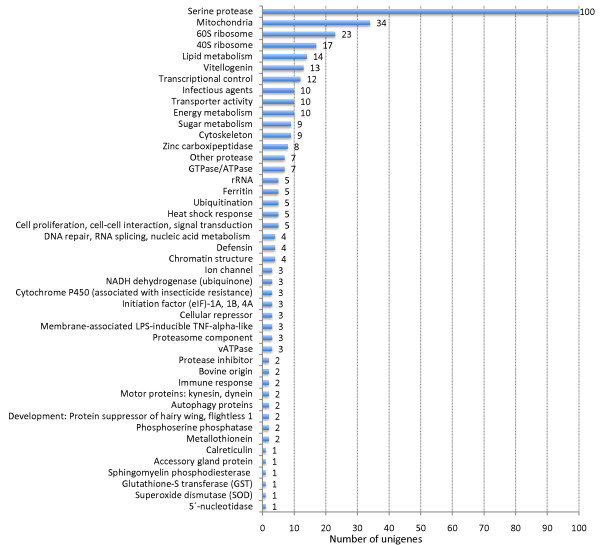
**Functional grouping of horn fly unigenes based on Gene Ontology (GO) molecular function assignments**.

**Figure 2:**
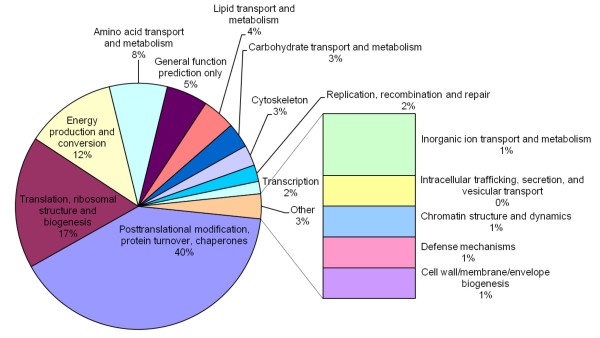
**Horn fly unigene assignment to Clusters of Orthologous Groups of proteins (COG; http://www.ncbi.nlm.nih.gov/COG)**.

BLAST searches to TrEMBL and Swiss-Prot databases assigned 367 proteins to molecular function Gene Ontology (GO) terms (Figure 1). One hundred unigenes (10%) containing 535 ESTs (25%) corresponded to serine proteases. Other molecular functions represented in the unigenes included those involved in cell metabolism, mitochondrial function, transcription and translation, transport, chromatin structure, vitellogenesis, cytoskeleton, DNA replication, cell response to stress and infection, cell proliferation and cell-cell interactions, intracellular trafficking and secretion, and development (Figure 1). Of the 367 unigenes with molecular function GO assignments, 184 could be assigned to Clusters of Orthologous Groups of proteins (COG) (Figure 2). The COG comprising posttranslational modification, protein turnover and chaperones contained 40% of proteins with COG assignments, followed by translation, ribosomal structure and biogenesis (17%) and energy production and conversion (12%) (Figure 2).

A relatively large set of 449 unigenes (45%) lacked any significant sequence similarity (Blast *E *values > 10^-5^) to any sequence available in the public databases. Of all the 543 unigenes with significant sequence similarity to previously published sequences, 88.8% were most similar to Diptera (*Drosophila *spp., *H. irritans*, *Musca domestica*, *Lucilia cuprina*, *Glossina *spp., *Stomoxys calcitrans*, *Aedes aepgypti*, *Sarcophaga *spp., *Phlebotomus papatasi*, *Chrysomyia bezziana*, *Anopheles gambiae*, *Ceratitis stricta*, *Trichopalpus fraterna*, *Automola atomaria*, *Nanna tibiella*, *Bactrocera dorsalis*, *Lutzomyia longipalpis*, *Eristalinus punctulatus*, and *Ophiomyia *sp.), 1.5% to other insect species (*Spodoptera frugiperda*, *Tribolium castaneum*, *Gryllus bimaculatus*, *Lonomia obliqua*, *Nasonia vitripennis*, and *Lymantria dispar*), 5.5% to other eukaryotic organisms, and 4.2% to microorganisms (Table 2).

**Table 2:** Distribution of annotated unigenes to different species.

Species	Total No. of unigenes	Ratio (%)
All organisms	543	100

Insects (Diptera)	482	88.8

*Drosophila *spp.	289	53.2

*H. irritans*	89	16.4

*Musca domestica*	22	4.0

*Lucilia cuprina*	16	2.9

*Glossina *spp.	15	2.8

*Stomoxys calcitrans*	14	2.6

*Aedes aepgypti*	11	2.0

*Sarcophaga *spp.	9	1.7

*Phlebotomus papatasi*	3	0.6

*Chrysomyia bezziana*	3	0.6

*Anopheles gambiae*	3	0.6

*Ceratitis stricta*	1	0.2

*Trichopalpus fraterna*	1	0.2

*Automola atomaria*	1	0.2

*Nanna tibiella*	1	0.2

*Bactrocera dorsalis*	1	0.2

*Lutzomyia longipalpis*	1	0.2

*Eristalinus punctulatus*	1	0.2

*Ophiomyia *sp.	1	0.2

Insects (Other)	8	1.5

*Tribolium castaneum*	2	0.4

*Gryllus bimaculatus*	2	0.4

*Spodoptera frugiperda*	1	0.2

*Lonomia obliqua*	1	0.2

*Nasonia vitripennis*	1	0.2

*Lymantria dispar*	1	0.2

Other eukaryotes	30	5.5

Microorganisms	23	4.2

Over 90% of best hits matched to insects. Only less than 10% of all best hits matched to other eukaryotes and microorganisms.

Thirteen unigenes assembled from 505 sequence reads, contained more than 20 ESTs, most probably representing transcripts with highest abundance in abdominal tissues of partially fed female horn flies (Table 3). As expected from the results of the annotation of the entire EST dataset, 10 (77%) of these unigenes corresponded to serine proteases (Table 3). The second largest group of ESTs was derived from mitochondrial transcripts (Table 3). The analysis of serine protease unigene sequences showed that although some of them may be paralogs (for example unigenes 1-2 and 3-5; Table 3), other probably reflect sequence polymorphisms within the horn fly population because they had 97%-98% nucleotide sequence identity (for example between unigenes 3-5; Table 3).

**Table 3:** Transcripts with highest abundance in abdominal tissues of partially fed female horn flies.

		Unigene annotation		
		
Unigene No. [GenBank accession number]	Number of clustered ESTs	Sequence identity	Clusters of Orthologous Groups of proteins (COG)	Molecular function Gene Ontology (GO) term
1 [HO004732]	21	*L. cuprina *clone sbsp9 serine proteinase	Posttranslational modification, protein turnover, chaperones	Secreted trypsin-like serine protease
2 [HO004733]	21	*L. cuprina *clone sbsp9 serine proteinase	Posttranslational modification, protein turnover, chaperones	Secreted trypsin-like serine protease
3 [HO004734]	22	*H. irritans *serine protease	Posttranslational modification, protein turnover, chaperones	Secreted trypsin-like serine protease
4 [HO004735]	25	*H. irritans *serine protease	Posttranslational modification, protein turnover, chaperones	Secreted trypsin-like serine protease
5 [HO004736]	25	*H. irritans *serine protease	Posttranslational modification, protein turnover, chaperones	Secreted trypsin-like serine protease
6 [HO004737]	26	*D. pseudoobscura *GA21163-PA (Dpse\GA21163)	Amino acid transport and metabolism	Zinc carboxypeptidase
7 [HO004738]	28	*H. irritans *serine protease mRNA	Posttranslational modification, protein turnover, chaperones	Secreted trypsin-like serine protease
8 [HO004739]	30	*L. cuprina *clone sbsp9 serine proteinase	Posttranslational modification, protein turnover, chaperones	Secreted trypsin-like serine protease
9 [HO004740]	33	*H. irritans *serine protease	Posttranslational modification, protein turnover, chaperones	Secreted trypsin-like serine protease
10 [HO004741]	37	*H. irritans *mitochondrion, complete genome		Mitochondria
11 [HO004742]	40	*H. irritans *serine protease	Posttranslational modification, protein turnover, chaperones	Secreted trypsin-like serine protease
12 [HO004743]	90	*H. irritans *serine protease	Posttranslational modification, protein turnover, chaperones	Secreted trypsin-like serine protease
13 [HO004744]	107	*H. irritans *mitochondrion, complete genome		Mitochondria

### Functional characterization of horn fly ESTs by RNAi

For functional genomics studies, selected unigene functional groups were used in RNAi experiments in female horn flies (Table 4). These groups included serine protease, protease inhibitor, vitellogenin (VTG), ubiquitination, ferritin (FER), vacuolar (H^+^)-ATPase (vATPase), proteasome component, immune response and 5'-nucleotidase (5'-NUC) ESTs and were selected based on their putative function in insect biology and previous results of RNAi experiments in other arthropods (see Discussion). As controls, ESTs with sequence identity to Nora virus and *Wolbachia *endosymbionts were selected (Table 4). The injection of these control dsRNAs did not affect horn fly mortality (β = -0.01, Wald Chi^2 ^= 0.01, P = 0.91) and oviposition (P > 0.05) when compared to buffer-injected flies in 14 independent RNAi experiments (Table 5), thus supporting their use as controls. Significant gene knockdown was obtained for at least one targeted unigene sequence on each group except for the serine protease group 1 in which significant gene expression silencing was not obtained for any of the unigenes included in the analysis (Table 5). For some sequences, gene knockdown was observed as early as 6 h post-injection (hpi) and lasted at least until 36 hpi (Table 5). For other sequences in groups 8 and 9, gene knockdown was not detected until after 12 hpi (Table 5). In most cases, gene expression silencing was higher than 70% when compared to the control group (Table 5).

**Table 4:** Horn fly unigene functional groups selected for RNAi.

Group N°	Functional group	**N° of unigenes**^**a**^	**Unigenes selected for RNAi**^**b**^
1	Serine protease	100	5, 10, 14, 19, 42, 90, 224, 230

2	Protease inhibitor	2	2_B12, 24_H02

3	Vitellogenin	13	7, 20, 37, 76, 89, 145, 176, 7_D07

4	Ubiquitination	5	84, 146, 4_E04, 5_G03, 7_B08

5	Ferritin	5	26, 39, 154, 156, 10_A09

6	vATPase	3	7_F08, 9_A08, 17_H03

7	Proteasome component	3	6_G04, 7_A04, 12_H09

8	Immune response	2	6_F11, 10_G05

9	5'-nucleotidase	1	13_D07

10 (negative control)	Infectious agents: Nora virus *Wolbachia *endosymbionts	3 3	191 2_E12

^a^Number of unigenes grouped into this category.

^b^When the number of unigenes in the functional group was greater than 5, unigenes were selected for RNAi to include all different genes present in this category. Unigenes GenBank accession numbers are shown in Table 6.

**Table 5:** Results of RNAi experiments in female horn flies.

**Group No.**^**a**^	Cumulative percent mortality			**Oviposition (eggs per survived fly)**^**b**^	**Expression silencing (average% ± SD) with respect to group 10 control**^**c**^				**dsRNA injected**^**d**^
				
	12 hpi	24 hpi	36 hpi		6 hpi	12 hpi	24 hpi	36 hpi	
1 (Serine protease)	57 ± 6	69 ± 11	78 ± 2	0.88 ± 0.23	ND	ND	57 ± 50 0 ± 0	ND	90 230

2* (Protease inhibitor)	73 ± 11	84 ± 7	94 ± 1	2.56 ± 1.80	ND	ND	83 ± 3**	ND	2_B12

3 (Vitellogenin)	55 ± 11	67 ± 8	74 ± 8	0.31 ± 0.9**	ND	ND	0 ± 0 0 ± 0 100 ± 2*	ND	7 20 76

4 (Ubiquitination)	42 ± 12	67 ± 11	83 ± 16	0.64 ± 0.63	ND	ND	92 ± 6* 70 ± 24 46 ± 43	ND	84 146 4_E04

5* (Ferritin)	24 ± 23	39 ± 37	47 ± 41	0.22 ± 0.17**	ND	ND	68 ± 13* 86 ± 86	ND	26 154

6* (vATPase)	17 ± 13	24 ± 11	34 ± 3	0.08 ± 0.05**	ND	ND	78 ± 6* 100 ± 1* 99 ± 8*	ND	7_F08 9_A08 17_H03

7 (Proteasome component)	64 ± 3	76 ± 8	84 ± 11	0.38 ± 0.03**	98 ± 2* 100 ± 6*	100 ± 2* 67 ± 9*	ND ND	0 ± 0 ND	7_A04 12_H09

8* (Immune response)	66 ± 7	ND	99 ± 8	0.23 ± 0.09**	100 ± 8* 0 ± 0	92 ± 5 0 ± 0	ND ND	96 ± 9* 98 ± 8*	6_F11 10_G05

9* (5'-nucleotidase)	50 ± 11	ND	91 ± 22	0.12 ± 0.09**	0 ± 0	98 ± 9*	ND	70 ± 7*	13_D07

10 (negative control)	45 ± 24	57 ± 30	64 ± 27	1.26 ± 0.90	---	---	---	---	191 2_E12

Injection buffer	46 ± 28	57 ± 30	65 ± 29	1.50 ± 1.01	0 ± 0	0 ± 0	0 ± 0	0 ± 0	None

^a^The average ± S.D. of two independent RNAi experiments for each of the test groups 1-9 and 14 experiments for the negative control and injection buffer groups is shown. One hundred flies were used on each RNAi experiment. Cumulative percent mortality was evaluated in female horn flies at 12, 24 and 36 hpi. Survival curves (temporal rates of mortality) were compared between different treatments and the control group 10 using Cox Proportional Hazards Survival Regression analysis (*P < 0.05). Abbreviation: ND, not determined.

^b^Oviposition data from test groups 1-9 and the injection buffer control were compared with the unrelated dsRNA-injected control group 10 by Student's t-test (**P < 0.005).

^c^For analysis of expression silencing, the mRNA levels of each knockdown gene were determined by real-time RT-PCR in 4 individual flies each at 6, 12 and 24 or 36 hpi. Percent expression silencing shown at different hpi corresponds to each individual gene targeted by dsRNA injection. The mRNA levels were normalized against horn fly 16S rRNA and the mean of the duplicate normalyzed Ct values from test dsRNA-injected groups 1-9 and in the injection buffer control were compared with the unrelated dsRNA-injected control group 10 by Student's t-test (*P < 0.05, **P < 0.005).

^d^Unigenes GenBank accession numbers are shown in Table 6.

**Figure 3:**
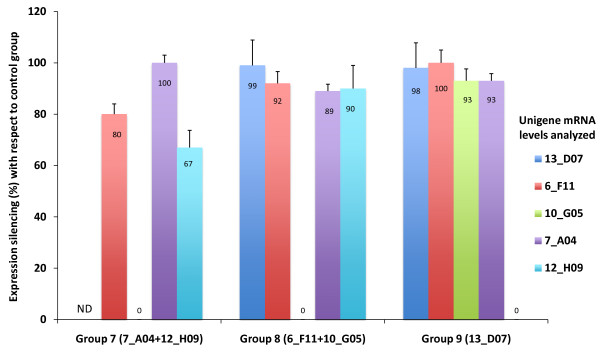
**Analysis of RNAi off-target effects in female horn flies**. Gene expression silencing (average % + SD) with respect to the control group was determined by real-time RT-PCR at 12 hpi in horn flies (N = 4) injected with different dsRNAs in three of the functional groups characterized by RNAi.

**Figure 4:**
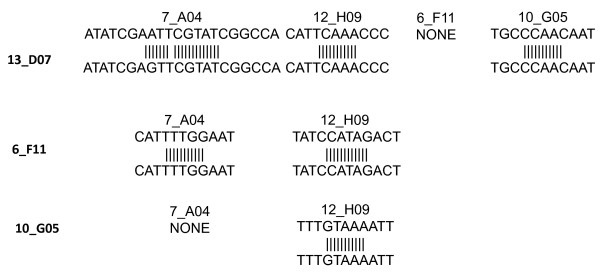
**Pairwise sequence alignment of dsRNA sequences showing homology regions ≥ 11 nucleotides**. Sequence alignments were done between the sequences used in the experiment described in figure 3. Abbreviation: ND, not determined.

To analyze RNAi off-target effects, the expression of genes not targeted by the injected dsRNA was analyzed at 12 hpi in functional groups 7-9 (Figure 3). The results showed that the expression of genes not targeted by the injected dsRNA was silenced in all three groups analyzed (Figure 3), thus suggesting RNAi off-target effects in horn flies. Pairwise sequence alignments identified regions with homology ≥ 11 bp in some sequences (Figure 4). However, only one region had 21 bp homology between unigene sequences 13_D07 and 7_A04 (Figure 4).

Injection of dsRNAs in the serine protease and ubiquitination functional groups did not affect fly mortality (β = 0.09, Wald Chi^2 ^= 2.68, P = 0.10 and β = 0.07, Wald Chi^2 ^= 3.60, P = 0.08, respectively) or oviposition (P > 0.05) when compared to controls (Table 5). The knockdown of a protease inhibitor gene (elastase) resulted in higher fly mortality (β = 0.60, Wald Chi^2 ^= 13.35, P = 0.0002) but did not affect oviposition (P > 0.05) when compared to controls (Table 5). VTG-2 and proteasome component genes knockdown did not affect fly mortality (β = 0.03, Wald Chi^2 ^= 1.42, P = 0.22 and β = 0.13, Wald Chi^2 ^= 2.59, P = 0.107, respectively) but significantly (P < 0.005) reduced oviposition (Table 5). When the expression of immune response and 5'-NUC genes was silenced, higher fly mortality (β = -0.46, Wald Chi^2 ^= 7.39, P = 0.006 and β = 0.35, Wald Chi^2 ^= 4.65, P = 0.03, respectively) and reduced oviposition (P < 0.005) were obtained when compared to controls (Table 5). Interestingly, knockdown of FER light chain and vATPase genes resulted in lower fly mortality (β = 0.21, Wald Chi^2 ^= 5.12, P = 0.02 and β=-0.16, Wald Chi^2 ^= 14.70, P = 0.0001, respectively), and 6 and 16-fold decrease in oviposition (P < 0.005) when compared to control dsRNA-injected flies (Table 5).

## Discussion

The effective control of horn fly infestations requires the design of new control strategies. Genomics and functional genomics studies are important to understand basic biological questions and to identify new targets for improved control strategies. Recently, gene expression analysis was reported in horn fly embryos, larvae and adult females [19,35]. However, this is the first report of functional genomics studies in this species.

ESTs sequenced and assembled in this study provided new sequence information for horn fly. The assembled unigenes without sequence similarity to sequences in public databases probably represented unique transcripts for horn fly or corresponded to proteins that have not yet been identified in related organisms due to incomplete genomic information. However, it cannot be excluded that the identified ESTs represent parts of known proteins whose similarities are located in parts of the sequence that are not covered by the analyzed ESTs.

The number of ESTs assembled into a certain unigene roughly reflected the relative abundance of corresponding mRNAs since the cDNA library from female horn flies used in this study was not normalized. We found that 100 unigenes, containing 25% of the ESTs, corresponded to serine proteases, indicating that this group represented the most abundantly expressed genes in abdominal tissues of partially fed female horn flies. In fact, the unigenes with the largest number of ESTs represented members of the serine protease family, thus suggesting that posttranslational modification and protein turnover were highly active in partially fed female flies.

The high proportion of ESTs present as singleton sequences when compared to contigs reflected a low diversity in our dataset, probably due to the presence of paralogs and sequence polymorphisms for some unigenes. In fact, sequence analysis of serine protease unigenes makes at this point difficult to discriminate between paralogs and ESTs representing sequence polymorphisms within the horn fly population.

RNAi was used to functionally characterize selected horn fly genes in adult female flies. To our knowledge, this is the first report of RNAi in horn flies. RNAi has been used to study gene function in insects and other arthropods [37,40-49] and to screen for candidate protective antigens in ticks [36-39]. Although with some fly mortality probably due to dsRNA injection with a Hamilton syringe, the RNAi method used here produced reproducible results in female horn flies. The failure to demonstrate gene knockdown for some sequences in serine protease and other functional groups studied could be due to unknown factors affecting RNAi in horn flies, because gene expression silencing did not occur until after 24-36 hpi for these genes or due to the existence of paralogs in these groups that affected the efficacy of gene knockdown. Gene silencing mediated by RNAi depends on short interfering RNAs (siRNAs) and micro RNAs (miRNAs). These RNAs have unique features, namely a defined size of 19-21 pb, and characteristic two-nucleotide single-stranded 3' overhangs and 5' monophosphate groups [50]. Although RNAi off-target effects were shown in horn flies, most sequence alignments resulted in homology regions of 11 bp only and in some cases no homology ≥ 11 bp was found. These results suggested differences in RNAi specificity and sensitivity, a fact that needs to be fully characterized to understand and efficiently use RNAi in horn flies and other organisms [37,48,51].

The aim of this study was to conduct a functional genomics study in female horn flies combining EST analysis with RNAi. Therefore, we will focus the discussion on unigene functional groups characterized by RNAi.

### Serine proteases

Serine proteases are a group of endopeptidases involved in several processes such as digestion, immune response, blood clotting and inflammation. In female horn flies, 10% of the assembled unigenes, containing more than 500 ESTs, were identified as serine proteases. In agreement with these results, Guerrero et al. [19] recently showed that serine proteases are differentially expressed in fly adult stages when compared to larvae. Significant gene knockdown was not obtained for any of the genes targeted by dsRNA injection in this group. Consequently, RNAi did not affect fly mortality or oviposition. In other arthropods, silencing of serine proteases expression by RNAi showed that these proteins are involved in blood digestion, oocyte maturation, development and immune response [40,42-44,46,52-55].

### Protease inhibitors

The protease inhibitor genes identified in female horn flies corresponded to serpins, inhibitors of serine proteases and thus involved in the same biological processes discussed before for serine proteases. A horn fly serine protease inhibitor gene was previously cloned and characterized, suggesting that these genes may be involved in the control of fly endogenous and pathogen proteases [56,57]. In mosquitoes, serpin RNAi affected insect immune response [58]. The elastase inhibitor gene knockdown significantly increased horn fly mortality at 12, 24 and 36 hpi. Thus, the effect of elastase inhibitor RNAi described here in horn flies may be the result of impaired fly protease control and/or the effect of increased susceptibility to persistent pathogen infections resulting from diminished immune response.

### Vitellogenin

VTGs constitute a multigene superfamily encoding for egg yolk precursor proteins expressed in the females of arthropods and other oviparous organisms [59]. In cockroaches, ants and ticks, knockdown of VTG receptor (VTG-R), essential for VTG uptake into developing oocytes, disrupts egg formation [41,60-62]. In honeybees, silencing of VTG expression by RNAi affects honeybee workers developmental behavior [63,64]. Similar to results of VTG-R knockdown in other arthropods, silencing of VTG-2 expression in horn flies reduced oviposition in 4-fold when compared to controls.

### Ubiquitination

Ubiquitination is a post-translational modification carried out by a set of enzymes that affect protein proteasomal degradation, stability, function, and intracellular localization [65]. In this functional group, horn fly genes involved in the ubiquitination pathway such as ubiquitin-1 (UBQ-1), UBQ-protein ligase, and UBQ hydrolase were included. In this group, only the UBQ-protein ligase expression was significantly silenced after RNAi. Although UBQ-protein ligase has been shown to regulate apoptosis in *Drosophila *[66], knockdown of this gene did not affect horn fly mortality or oviposition. Thus, it may be possible that the phenotype resulting from silencing the UBQ-protein ligase expression was not evident in horn flies under our experimental conditions. Additionally, knockdown of other ubiquitination genes may be required in order to have a significant effect on horn fly mortality and oviposition. For example, UBQ knockdown in ticks causes mortality and reduces oviposition and *Anaplasma marginale *infection/multiplication in the guts [39,67].

### Ferritin

FER is the main protein for intracellular iron storage and consists of 2 types of subunits, a heavy (ferroxidase sites) and a light chain (nucleation sites) [68]. FER light (3 unigens containing 5 ESTs) and heavy (2 unigenes containing 4 ESTs) chains were not among the most abundant ESTs identified in female horn flies. However, Guerrero et al. [35] found FER light chain as one of the most abundant transcripts in horn fly larvae. These results suggested differences in the FER expression between horn fly larvae and adult females. FER light chain knockdown in horn flies significantly reduced oviposition (6-fold with respect to controls), but surprisingly fly mortality was reduced when compared to controls. In ticks, FER RNAi reduces not only oviposition but also feeding and *A. marginale *infection levels in IDE8 cells [67,68].

### vATPase

vATPase is a multisubunit enzyme that mediates acidification of eukaryotic intracellular organelles and has been shown to be required for the normal function of the Golgi complex, endoplasmic reticulum, vacuoles and endocytotic and exocytotic vesicles [69]. vATPase was also implicated in immunity [70]. Guerrero et al. [35] identified vATPase as one of the most abundant transcripts in horn fly larvae. However, in adult females, only 3 ATPase unigenes were assembled with one EST each, those suggesting like previously for FER, differences in the vATPase expression between horn fly larvae and adult females. Genetic knockout of vATPase subunits resulted in lethal phenotypes in fruit flies (*Drosophila melanogaster*), flour beetles (*T. castaneum*), pea aphids (*Acyrthosiphon pisum*), and tobacco hornworms (*Manduca sexta*) [69,71] and reduced influenza virus replication in *Drosophila *cells [72]. RNAi of vATPase expression in ticks resulted in testis and salivary gland degeneration, suggesting a role for this molecule in the function of these organs [73] and reduced *A. marginale *infection in *Dermacentor variabilis *tick guts but not pathogen multiplication in IDE8 tick cells [67]. vATPase knockdown in horn flies resulted in 16-fold reduction in oviposition but, as with FER light chain, fly mortality was reduced when compared to controls. These results suggested that despite the important function of vATPase in all arthropods, developmental stage-specific and species-specific differences might exist that could explain the results obtained after gene knockdown in horn flies.

### Proteasome component

Proteasomes are large protein complexes involved in protein proteolysis that are functionally related to ubiquitination and thus essential for eukaryotic cells [74]. Experiments in *D. melanogaster *showed that knockdown of proteasome subunits leads to increased levels of ubiquitin conjugates, cell cycle defects, DNA overreplication, and apoptosis [74,75]. In tick cells, 26S proteasome knockdown resulted in lower *A. marginale *infection levels when compared to controls but did not affect tick survival, feeding and reproduction [67]. However, based on the essential proteasome function in eukaryotic cells, it was not surprising to observe a decrease in oviposition in horn flies injected with proteasome components dsRNAs targeting proteasome subunit beta (two unigenes) and proteasome maturation protein (one unigene). As previously shown in *D. melanogaster *[74,75], proteasome subunits knockdown in horn flies may affect cell cycle and DNA replication thus resulting in reduced oviposition.

### Immune response

Innate immune response is essential for insect survival. Only two unigens were assembled into this category and knockdown in female horn flies. Assembled unigenes encoded for putative T-cell immunomodulatory protein and RNAse L inhibitor. Silencing of these genes resulted in higher horn fly mortality and lower oviposition when compared to controls. These RNAi results may be due to an effect of gene knockdown on increased susceptibility to persistent pathogen infections resulting from impaired immune response in horn flies. Knockdown of immune response genes may affect the mechanisms involved in the control of persistent infections such as those caused by Nora virus and *Wolbachia *spp. [76-78], which could affect horn fly mortality and ovisposition. RNAi knockdown of immune response genes in other arthropods results in increased mortality and higher pathogen infection levels [79-81].

### 5'-nucleotidase

5'-NUC and other ectonucleotidases control the levels of extracellular nucleotides and nucleosides that act as signaling molecules involved in a wide spectrum of biological effects [82]. 5'-NUC is commonly expressed in the salivary glands of blood-sucking ectoparasites [83-88]. Herein, as previously shown in ticks [36], 5'-NUC knockdown resulted in higher fly mortality and lower oviposition when compared to controls. As in other organisms, these results suggested an essential function for 5'-NUC in horn fly females.

## Conclusions

In summary, a cDNA library was constructed from whole abdominal tissues collected from partially fed adult female horn flies and 2,160 high quality ESTs were sequenced and assembled into 992 unigenes (178 contigs and 814 singlets) representing molecular functions such as serine proteases, cell metabolism, mitochondrial function, transcription and translation, transport, chromatin structure, vitellogenesis, cytoskeleton, DNA replication, cell response to stress and infection, cell proliferation and cell-cell interactions, intracellular trafficking and secretion, and development. A method was developed for RNAi that produced reproducible results in horn flies. Functional analyses by RNAi showed the effect of some genes on horn fly mortality and oviposition. These results advanced the molecular characterization of this important ectoparasite and suggested candidate protective antigens for the development of vaccines for the control of horn fly infestations. Based on RNAi results, some of the candidate antigens to be considered for cattle vaccination experiments against horn flies include those within VTG, immune response and 5'-NUC functional groups.

## Methods

### Rearing of horn flies

*H. irritans *were reared under laboratory conditions as reported by Schmidt et al. [89]. A horn fly colony was established with flies originally collected in a cattle farm close to Ciudad Victoria, Tamaulipas, Mexico. About 2,000 flies were collected from 2 infested animals and transported in a 20 × 30 cm mosquito netting aluminum cage. Flies were allowed to lay eggs over a water container during 12 h. Eggs were collected and incubated into fresh bovine feces during 5 days. Pupae were collected and placed in Petri dishes located inside mosquito netting aluminum cages for molting into adult flies. After molting, flies were fed twice a day using pieces of cotton impregnated with fresh defibrinated bovine blood obtained from a naive cow. All the horn fly developmental phases were kept under a photoperiod of 12 h light: 12 h darkness at 28-32°C and 70-80% relative humidity [89].

### Analysis of expressed sequence tags (ESTs) in adult female horn flies

Total RNA was isolated from whole abdominal tissues collected from 1,500 partially fed adult female horn flies using Trizol (Sigma, St. Louis, MO, EUA). The cDNA library was synthesized using the SMART™ cDNA Library Construction Kit (Clontech, Mountain View, CA, USA) at Creative Biolabs (Port Jefferson Station, NY, USA; http://www.creativebiolabs.com). cDNAs were cloned into the pBluescript II SK vector (Agilent Technologies, Inc., Santa Clara, CA, USA). The library had more than 1×10^6 ^primary clones, with >90% recombinants with inserts >500 bp (average cDNA length >1,000 bp). A total of 2,462 ESTs were 5' sequenced (Creative Biolabs). The cDNA Annotation System software (CAS; Bioinformatics and Scientific IT Program (BSIP), Office of Technology Information Systems (OTIS), National Institute of Allergy and Infectious Diseases (NIAID), Bethesda, MD, USA) http://exon.niaid.nih.gov was used for automated sequence clean up, assembly, blasting against multiple sequence databases (ncbi non-redundant nucleotide and protein sequence databases, *H. irritans *EST sequences [35] and databases of mosquito- and tick-specific sequences http://www.ncbi.nlm.nih.gov/; http://www.vectorbase.org/index.php) and Gene Ontology (GO) assignments. Comparison with the ncbi Clusters of Orthologous Groups of proteins (COG; http://www.ncbi.nlm.nih.gov/COG) was also performed. Nucleotide sequences were aligned using the program AlignX (Vector NTI Suite V 5.5, InforMax, North Bethesda, MD, USA). Gene sequences were deposited in the GenBank with accession numbers HO000420-HO001165 and HO004499-HO004744.

### RNAi in adult female horn flies

Oligonucleotide primers (pBLUET75: 5'-*TAATACGACTCACTATAGGGTACT*TCGAGGTCGACGGTATCGAT-3' and pBLUET73: 5'-*TAATACGACTCACTATAGGGTACT*CAATTAACCCTCACTAAAGGGA-3') were synthesized specific for vector DNA sequences flanking the horn fly cDNA insert and containing T7 promoter sequences (in italics) for *in vitro *transcription and synthesis of dsRNA. PCR reactions were performed from individual or pooled cDNA clones (when more than one unigene was included in the functional group analyzed; Table 2) using the Access RT-PCR system (Promega, Madison, WI, USA) in a 50 μl reaction mixture. The resultant amplicons were purified using the Wizard 96-well PCR purification system (Promega). *In vitro *transcription and purification of dsRNA was done using the Megascript RNAi kit (Ambion, Austin, TX, USA). The dsRNA was quantified by spectrometry. Adult partially fed female flies were injected with approximately 0.1 μl of dsRNA (1 × 10^9^-1 × 10^11 ^molecules per μl) in the abdominal segment. The injections were done with a Hamilton syringe with a 1 inch, 33 gauge needle. Control flies were injected with unrelated dsRNA or injection buffer (10 mM Tris-HCl, pH 7, 1 mM EDTA) (Table 5). One hundred flies were used in each group. After injection with dsRNA, female flies were kept in petri dishes for one hour and then transferred to wired 20 × 30 cm boxes. Flies were fed using impregnated cotton with fresh defibrinated blood obtained from a naive cow and reared as described before. Fly mortality was evaluated at 12, 24 and 36 hpi. Survival curves (temporal rates of mortality) were compared between different treatments and controls using Cox Proportional Hazards Survival Regression analysis (SPSS Inc., Chicago, IL, USA). Oviposition (number of eggs per survived fly) was also evaluated and the results in test dsRNA-injected groups and in the injection buffer control were compared with the unrelated dsRNA-injected control group by Student's t-test (P = 0.05).

Gene expression silencing was evaluated in 4 individual flies each at 6, 12 and 36 or 24 hpi. The mRNA levels of each knockdown gene were determined using sequence-specific oligonucleotide primers (Table 6) and the iScript One-Step RT-PCR Kit with SYBR Green and the iQ5 thermal cycler (Bio-Rad, Hercules, CA, USA) following manufacturer's recommendations. A dissociation curve was run at the end of the reaction to ensure that only one amplicon was formed and that the amplicon denatured consistently in the same temperature range for every sample [90]. The mRNA levels were normalized against horn fly 16S rRNA (Table 4) using the genNorm method (ddCT method as implemented by Bio-Rad iQ5 Standard Edition, Version 2.0) [91]. In all cases, the mean of the duplicate values was used and normalyzed Ct values from test dsRNA-injected groups and in the injection buffer control were compared with the unrelated dsRNA-injected control group by Student's t-test (P = 0.05).

**Table 6:** Primer sets and real-time RT-PCR conditions used for analysis of selected *H. irritans *ESTs.

Unigene ID (Genbank accession number)	Gene description	Upstream/downstream primer sequences (5'-3')	PCR annealing conditions
13_D07 (HO000820)	5'-nucleotidase	AGTGGACAAATGTCCCGAAG AGCATTGGGGTTTGAATGAG	55°C, 30 s

6_F11 (HO000609)	T-cell immunomodulatory protein	CCGGTGACTTTGATGGAGAT GATAATGGCTCCCCTTTGGT	55°C, 30 s

10_G05 (HO000738)	RNase L inhibitor	GCCGATCGTGTTATTGTCCT CCGGATCGTTTTTGTTCAGT	55°C, 30 s

7_A04 (HO000619)	Proteasome subunit beta	CAGGCGAGGTCCATTATTGT AGTGCGCGACCTCAAGTAGT	55°C, 30 s

12_H09 (HO000808)	Proteasome maturation protein	GAGGAATCGTGAGGGTTTGA ACATGGGGTTGTCGGATAAA	55°C, 30 s

7_F08 (HO000644)	vATPase subunit d	TGTTTTTCCGTCACCAGTCA GGCACAAACCCTCCAAGTAA	60°C, 30 s

9_A08 (HO000679)	vATPase subunit f	TGTTGGATTCTTGCTTGGTG GGCACTGGTGATGTATGTGC	60°C, 30 s

17_H03 (HO001053)	vATPase proteolipid subunit	GTCCAGCCAGACTGTGATGA AATCAATCGCGGACAAAAAC	60°C, 30 s

26 (HO004524)	Ferritin light chain	TGATCATGTTGAACCCGAGA CGGCTGGTCAATTTCTTGAT	60°C, 30 s

154 (HO004652)	Ferritin heavy chain	GTTGTTGCCCCTGCTGTATT TGAAAAGTGGGCTCCCATAG	60°C, 30 s

84 (HO004582)	Ubiquitin-protein ligase	TCGCATCTGTTTGGATGTGT CGGGAAAACTTTTGAGTCCA	60°C, 30 s

146 (HO004644)	Ubiquitin family (UBQ-1)	CCCGACCAACAACGTTTAAT CGACGAAGACGGTGAATTTT	60°C, 30 s

4_E04 (HO000538)	Ubiquitin carboxyl-terminal hydrolase	AGCCAGAGATGTTGGAATGG TCGATGTAAATTGCCGCATA	60°C, 30 s

7 (HO004505)	Vitellogenin 3	GAGCTTTTTGCGTTGTAGCC ACAAAAGTGGGAGCAACACC	60°C, 30 s

20 (HO004518)	Vitellogenin 1	GAGCTTTTTGCGTTGTAGCC ACAAAAGTGGGAGCAACACC	60°C, 30 s

76 (HO004574)	Vitellogenin 2	ACGGCCGGTTGTGAGATTAT AGCATCTTTTTCGGTCTTGC	60°C, 30 s

2_B12 (HO000479)	Elastase inhibitor	CAAGGGTGAATGGGAAAAGA TAAAGGCCTTCACGTTCCTG	60°C, 30 s

90 (HO004588)	Serine protease, midgut specific trypsin, secreted	TGCGTTATATTCCGTTGGTG CTTTGTCAACGGCATAAGCA	60°C, 30 s

230 (HO004728)	Serine protease	TGGCTACAATGAATGCAAGC GGTTAGCACCAGGGAACGTA	60°C, 30 s

(FJ025436)	*H. irritans *16S rRNA	TTTAAATGGCCGCAGTATCC GATTTATAGGGTCTTCTCGTCTTTT	60°C, 30 s

## Authors' contributions

LT reared horn flies, prepared RNA samples, did the RNAi experiments and helped with drafting the manuscript. CA participated in coordination of the study, RNAi experiments and drafting of the manuscript. NA, RCG and CA conducted the real-time RT-PCR analyses. RCG helped with drafting the manuscript. JF participated in design and coordination of the study, analyzed EST and RNAi data and drafted the manuscript. RRC and HQR participated in coordination of the study. All authors read and approved the final manuscript.

## Supplementary Material

Additional file 1 **Table S1**. Assembling and analysis of *H. irritans *high quality ESTs. Click here for file
